# German population norms of the preference to solitude scale and its correlates

**DOI:** 10.1371/journal.pone.0303853

**Published:** 2024-05-21

**Authors:** André Hajek, Angelina R. Sutin, Martina Luchetti, Yannick Stephan, Karl Peltzer, Antonio Terracciano, Hans-Helmut König

**Affiliations:** 1 Department of Health Economics and Health Services Research, University Medical Center Hamburg-Eppendorf, Hamburg Center for Health Economics, Hamburg, Germany; 2 Florida State University College of Medicine, Tallahassee, FL, United States of America; 3 Euromov, University of Montpellier, Montpellier, France; 4 Faculty of Public Health, Department of Health Education and Behavioral Sciences, Mahidol University, Bangkok, Thailand; 5 Department of Psychology, University of the Free State, Bloemfontein, South Africa; 6 Department of Psychology, College of Medical and Health Science, Asia University, Taichung, Taiwan; University of Campinas - UNICAMP, BRAZIL

## Abstract

**Aim:**

Our first aim was to present norm values for the Preference for Solitude Scale by sex, age, and other sociodemographic groups. Our second aim was to evaluate the correlates of preference for solitude.

**Methods:**

Data were collected in August/September 2023 from a sample of individuals (N = 5000) living in Germany aged 18 to 74 years (ensuring representativeness in terms of sex, age group and federal state for the German general adult population). The established and valid Preference for Solitude Scale (range 0 to 12, with higher values reflecting a stronger preference for solitude) was used to quantify the preference for solitude. Norm values were provided by sex and age groups. Multiple linear regressions were used to examine the correlates of preference for solitude.

**Results:**

Average preference for solitude score was 7.6 (SD = 3.0; 0 to 12). The average score was 7.3 (SD = 3.0) among males and 7.9 (SD = 2.9) among females. Regressions showed that a stronger preference for solitude was associated with being female (β = .51, p < .001), being older (e.g., being 40 to 49 years compared to 18 to 29 years, β = .85, p < .001), being single (e.g., divorced compared to being single, β = -.78, p < .01), higher level of education (secondary education compared to primary education, β = .43, p < .01), never been a smoker (e.g., daily smoker compared to never smokers, β = -.61, p < .001), absence of alcohol consumption (e.g., drinking once a week compared to never drinking, β = -1.09, p < .001), no sports activity (e.g., 2–4 hours per week compared to no sports activity, β = -.60, p < .001), poorer self-rated health (β = .28, p < .001) and more depressive symptoms (β = .05, p < .001). Sex-stratified regressions yielded similar results.

**Conclusion:**

Norm values provided in this study can be used as a benchmark for comparison with other countries and can guide further research dealing with preferences for solitude. We demonstrated the importance of several sociodemographic factors (e.g., marital status), lifestyle-related factors (e.g., sports activity), and health-related factors (e.g., depressive symptoms) for the preference for solitude. Such knowledge about the correlates of preference for solitude may help to characterize them. This is essential to ensure a good balance between social interaction and being alone. This is important because preference for solitude is associated with poor self-rated health and depression, but also with healthy behaviors such as abstaining from smoking and drinking

## 1 Introduction

A preference for solitude is defined as the tendency to spend and enjoy time alone [1]. Interest in this construct has gained attention in recent years, particularly since the beginning of the Covid-19 pandemic [2,3] and the concerns related to loneliness and declining social connections [4]. Preference for solitude may result from intrinsic characteristics and life circumstances and can have both positive and negative correlates (e.g., mental health). Some people might find it easier to relax, concentrate, reflect on their life, connect with nature, or engage in creative activities when alone. A preference for solitude could also develop in reaction to stressful or unfulfilling social interactions. Such preference could differ for men and women, who are thought to have different affiliative traits [5], and evolve as people get older. For example, a recent study showed that extraverted narcissism is associated with aversion to solitude [6]. A trend to a stronger preference for solitude was recently observed among Chinese adolescents (particularly among urban adolescents) [7]. Another study showed the potential negative correlates of this preference, including harmful interpersonal consequences because individuals with a stronger preference for solitude had a higher likelihood of experiencing ostracism and belief that engaging with individuals who strongly preferred solitude would be unpleasant for both interaction partners [8]. A further study showed that a greater preference for solitude was associated with higher odds of suicidal ideation and self-harm among adolescents in Japan (city of Tsu and Kochi Prefecture) [9].

Even though there have been more studies on being alone since the beginning of the pandemic, the preference for solitude (e.g., in contrast to research on isolation and loneliness) has hardly been studied at all. The few existing studies are often restricted to selective and small samples–or focused on certain groups such as adolescents/young adults (e.g., [10,11]). Burger [1], for example, provided norms for undergraduate college students in the United States (n = 103): 5.37 (SD = 2.45) among male students and 4.52 (SD = 2.61) among female students (combined: 4.87, SD = 2.57). There is thus a need for larger and more diverse samples to provide both norms and identify correlates of preference for solitude among the general adult population. To that end, we use a large sample of German adults to provide norm values for the Preference for Solitude Scale by sex and age groups and other important sociodemographic groups. Moreover, we aim to investigate the correlates of preference for solitude among the general adult population in Germany. Such knowledge can enrich our understanding of individual differences in social behavior, which is important because a greater preference for solitude may have consequences for well-being (which can be either positive or negative, depending on the outcome [12]) and mental health outcomes [9].

## 2 Methods

### 2.1 Sample

We used data from a study of 5,000 individuals living in Germany, ranging in age from 18 to 74 years old. Data collection occurred during August and September 2023. Bilendi, a market research firm certified with ISO 26362, handled participant recruitment for this study. Selection was done from an online pool using quotas to achieve a balanced representation across age, sex, and federal states, which mirrors the corresponding demographics of the general adult population in Germany.

All participants gave written informed consent to participate in the study before the survey began (by agreeing to the online consent form before the survey began), which is a standard procedure for online surveys. Additionally, the study obtained approval from the Local Psychological Ethics Committee at the University Medical Center Hamburg-Eppendorf (LPEK-0629).

### 2.2 Preference for solitude

To quantify the preference for solitude, we used the German version [13] of the Preference for Solitude Scale [1]. For each pair of statements, participants were asked to select the statement that best described them. Whenever neither statement described them well, or both statements described them somewhat, they should choose the statement that applied to them more often (or better). One example pair is:

1a) I enjoy being around people.
1b) I enjoy being by myself.

Each pair of statements, the statement preferring others was coded as 0 (reflecting a weak preference for solitude; example statement 1a) and the statement preferring being alone was coded as 1 (reflecting a strong preference for solitude; example statement 1b). The final count score ranged from 0 to 12, with higher values indicating a stronger preference for solitude. The former study showed that the German version of the Preference for Solitude Scale is internally consistent (Cronbach’s alpha was .77) and valid [13]. A good test-retest reliability (.75) has also been demonstrated [13].

### 2.3 Correlates

Potential correlates of preference for solitude were selected based on former research (e.g., [14]). Sociodemographic correlates included sex (males; females; diverse), age group (18 to 29 years; 30 to 39 years; 40 to 49 years; 50 to 59 years; 60 to 74 years), marital status (single; divorced; widowed; living together: married or in partnership; living separately: married or in partnership), education (according to the CASMIN-classification [15]: primary education; secondary education; tertiary education), labor force participation (full-time employed; retired; other), region (West Germany; East Germany), and migration background (no; yes). Lifestyle-related correlates were considered: smoking behavior (yes, daily; yes, occasionally; no, not anymore; never been a smoker), alcohol consumption (daily; several times a week; once a week; one to three times a month; less often; never), and frequency of sports activities (no sports activity; less than one hour per week; 1 to 2 hours per week; 2 to 4 hours per week; more than 4 hours per week). Additionally, we considered health-related correlates: self-rated health (single-item, ranging from 1 = very good to 5 = very poor), count of chronic conditions (Sleep disorder; Thyroid disease; Diabetes; Asthma; Heart disease [also heart failure, cardiac insufficiency]; Cancer; Stroke; Migraine; High blood pressure; Dementia; Joint disease [also arthrosis, rheumatism]; Chronic back problems; Burnout; Other illness), and depressive symptoms. Depressive symptoms were assessed using the 9-item Patient Health Questionnaire-9 (PHQ-9) [16]. A total score ranging from 0 to 27 was calculated, with higher scores indicating more depressive symptoms. In sensitivity analysis, depressive symptoms were replaced by anxiety symptoms (due to collinearity, they were not included in one model). Anxiety symptoms were measured using the 7-item Generalized Anxiety Disorder-7 (GAD-7) [17]. The total sum score ranged from 0 to 21, with higher scores indicating more anxiety symptoms.

### 2.4 Statistics

To address the first aim of this study, the characteristics of the sample and population norms for the sociodemographic subgroups (i.e., sex, age group, marital status, education, migration background, employment status, region) were computed. To address the second aim of this study, the correlates of the preference for solitude were investigated using multiple linear regressions with robust standard errors. Using coefplot [18], the regression coefficients are also displayed. Depressive symptoms were replaced by anxiety symptoms in a sensitivity analysis because they could not be tested simultaneously due to collinearity issues. Additional analyses tested sex-stratified regressions (males; females). No missing values were present (due to the forced-choice format). Stata 18.0 (Stata Corp., College Station, Texas) was used in this present study. The statistical significance was set at p < 0.05.

## 3 Results

### 3.1 Sample characteristics and population norms

Characteristics of the sample are presented in Table 1. The average age was 46.9 years (SD = 15.3 years, range 18 to 74 years) and 50.8% female. Additional details are provided in Table 1.

**Table 1:** Sample characteristics (n = 5,000).

Variables	Mean (SD) / n (%)
Sex	
Men	2451 (49.0)
Women	2540 (50.8)
Diverse	9 (0.2)
Age (in years)	46.9 (15.3)
Age group	
18–29 years	880 (17.6)
30–39 years	887 (17.7)
40–49 years	874 (17.5)
50–59 years	1129 (22.6)
60–74 years	1230 (24.6)
Marital status	
Single	1333 (26.7)
Divorced	403 (8.1)
Widowed	160 (3.2)
Living together: Married/Partnership	2893 (57.9)
Living separated: Married/Partnership	211 (4.2)
Education	
Primary education	533 (10.7)
Secondary education	2987 (59.7)
Tertiary education	1480 (29.6)
Employment status	
Full-time employed	2418 (48.4)
Retired	1000 (20.0)
Other	1582 (31.6)
Region	
West Germany	4210 (84.2)
East Germany	790 (15.8)
Migration background	
No	4449 (89.0)
Yes	551 (11.0)
Smoking status	
Yes, daily	1184 (23.7)
Yes, occasionally	516 (10.3)
No, not anymore	1366 (27.3)
Never been a smoker	1934 (38.7)
Alcohol consumption	
Daily	275 (5.5)
Several times a week	1030 (20.6)
Once a week	898 (18.0)
1–3 times a month	828 (16.6)
Less often	1093 (21.9)
Never	876 (17.5)
Frequency of sports activity	
No sports activity	1255 (25.1)
Less than one hour per week	917 (18.3)
1–2 hours per week	1202 (24.0)
2–4 hours per week	889 (17.8)
More than 4 hours per week	737 (14.7)
Self-rated health (from 1 = very good to 5 = very poor)	2.4 (0.8)
Number of chronic conditions (from 0 to 14, higher scores reflect a higher number of chronic conditions)	1.5 (1.6)
Depressive symptoms (PHQ-9; ranging from 0 to 27, whereby higher scores reflect more depressive symptoms)	5.7 (5.4)
Anxiety symptoms (GAD-7; ranging from 0 to 21, whereby higher scores reflect more anxiety symptoms)	4.2 (4.6)

The population norms for the Preference for Solitude Scale are shown in Table 2. The average preference for solitude score was 7.6 (SD = 3.0) on a scale from 0 to 12. The average score was 7.3 (SD = 3.0) among men and 7.9 (SD = 2.9) among women (Cohen’s d = |.20|). The average preference for solitude score varied between the age group (e.g., 8.1 (SD = 2.9) among individuals aged 50 to 59 years vs. 7.2 (SD = 2.8) among individuals aged 18 to 29 years, Cohen’s d = |.32|). Similarly, single participants had an average preference for solitude score of 8.1 (SD = 2.9), whereas individuals living together with their partner or spouse had an average score of 7.3 (SD = 3.0, Cohen’s d = |.27]). Individuals with a tertiary education had a comparatively lower average preference for solitude (7.2, SD = 3.0) compared to lower educated individuals. Full-time employed individuals had an average preference for solitude score of 7.3 (SD = 3.0), whereas retired individuals had an average score of 8.0 (SD = 3.0, Cohen’s d = |.23|). The average preference for solitude was particularly high among unemployed/job-seeking individuals (8.2, SD = 3.0; not shown in Table 2). No difference in the preference for solitude was found based on region or migration background.

**Table 2:** Norm values for preference for solitude (also among several groups).

	Preference for solitude	p-value
Total sample	7.6 (3.0)	
Sex		< .001
Male	7.3 (3.0)	
Female	7.9 (2.9)	
Diverse	7.2 (3.8)	
Age group		< .001
18 to 29 years	7.2 (2.8)	
30 to 39 years	7.0 (3.0)	
40 to 49 years	7.7 (3.1)	
50 to 59 years	8.1 (2.9)	
60 years and older	7.7 (3.0)	
Marital status		< .001
Single	8.1 (2.9)	
Divorced	8.0 (3.3)	
Widowed	7.8 (3.0)	
Living together: Married/Partnership	7.3 (3.0)	
Living separated: Married/Partnership	7.7 (2.9)	
Education		< .001
Primary education	7.6 (3.0)	
Secondary education	7.8 (2.9)	
Tertiary education	7.2 (3.0)	
Migration background		.20
No	7.6 (3.0)	
Yes	7.5 (2.9)	
Employment status		< .001
Full-time employed	7.3 (3.0)	
Retired	8.0 (3.0)	
Other	7.8 (3.0)	
Region		.65
West Germany	7.6 (3.0)	
East Germany	7.6 (3.0)	

Notes: Independent t-tests or oneway anovas were performed, as appropriate.

### 3.2 Regression analysis

**Fig 1:**
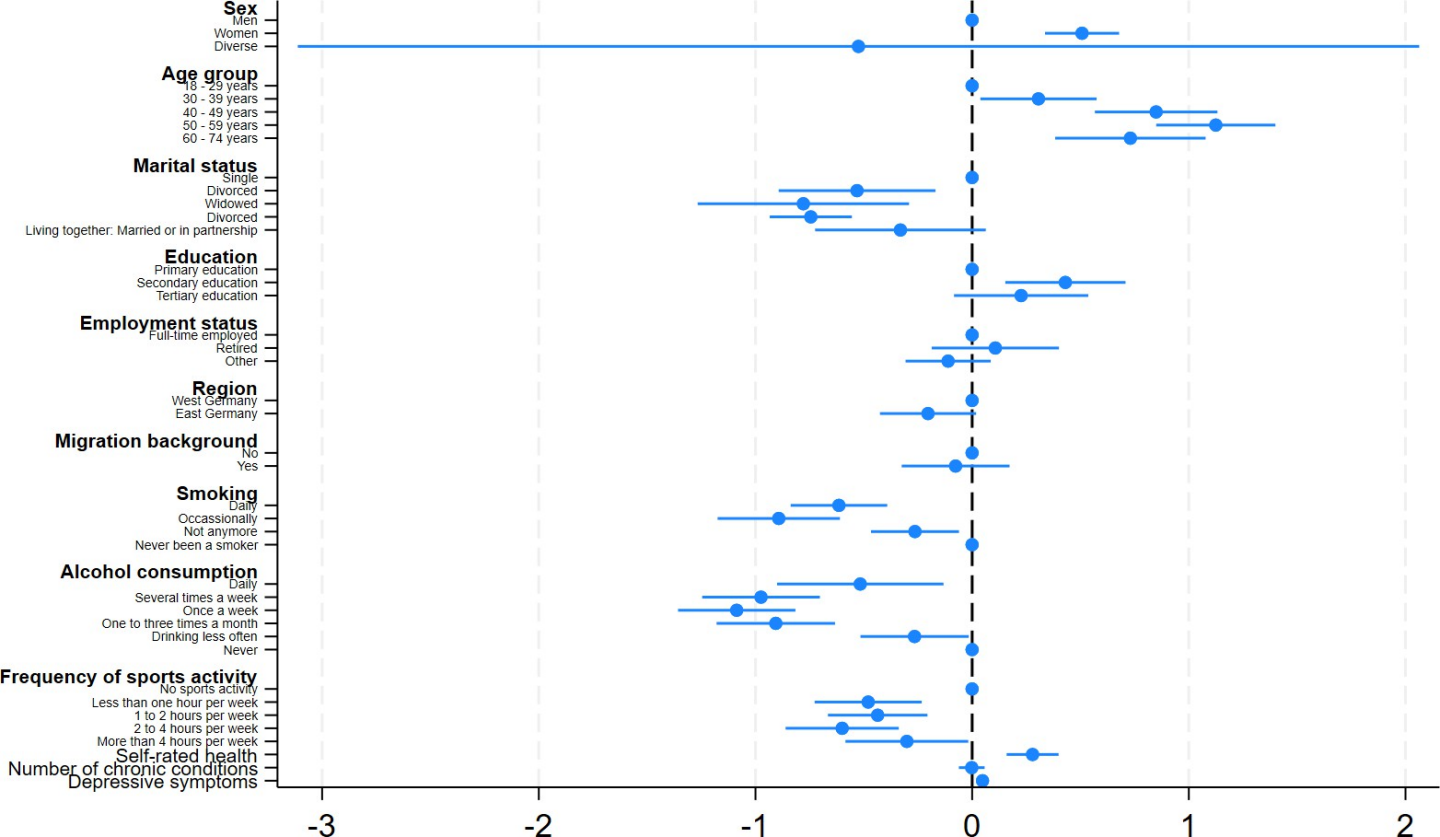
Results of multiple linear regressions (among the total sample).

**Fig 2:**
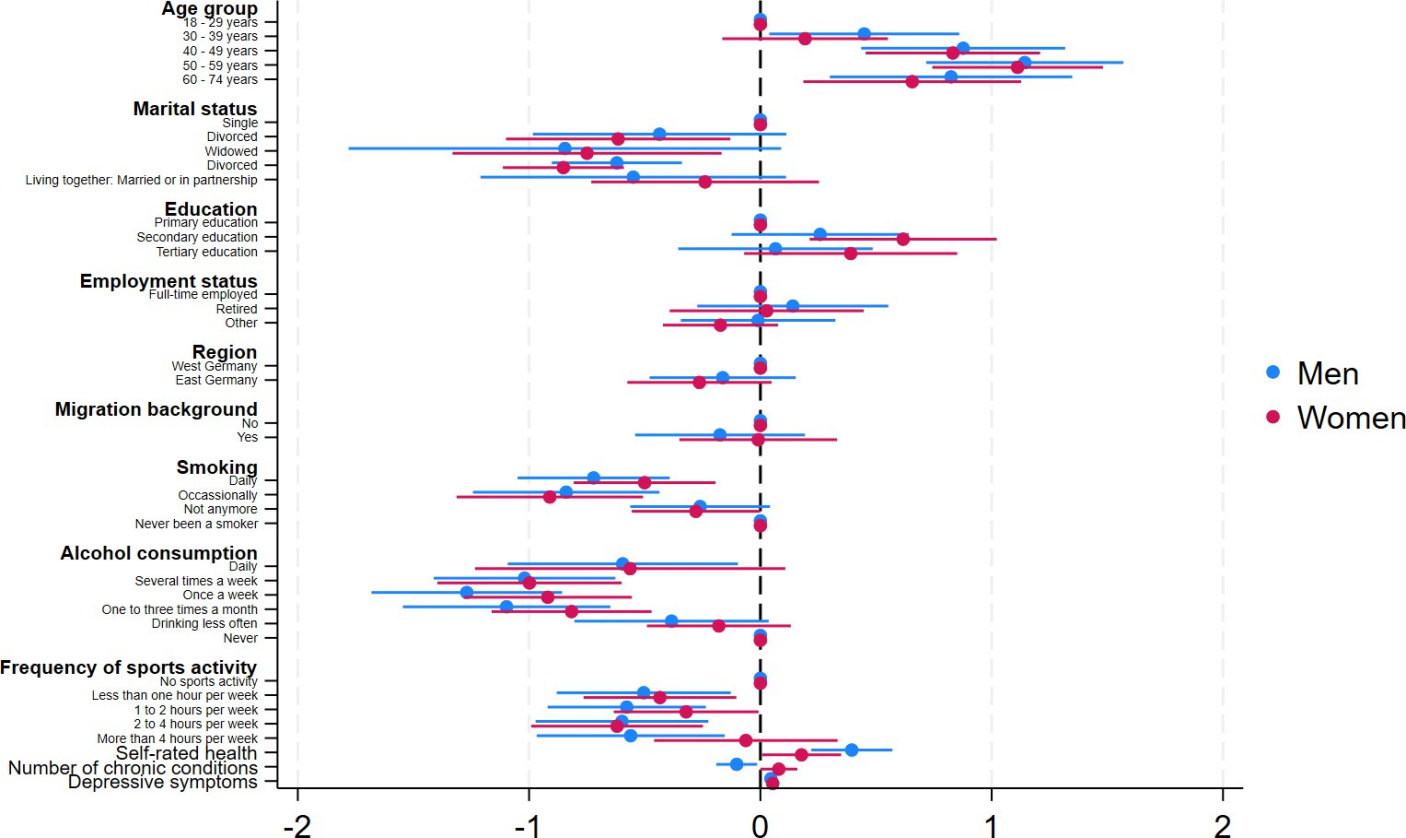
Results of multiple linear regressions (stratified by sex).

Results of the multiple linear regression with preference for solitude as the outcome are in Table 3 (the coefficients are also graphically displayed in Figs 1 and 2 to ease the readability). The regression showed that a stronger preference for solitude was associated with being female (β = .51, p < .001), being older (e.g., 50 to 59 years compared to 18 to 29 years, β = 1.12, p < .001), being single (e.g., divorced compared to being single, β = -.78, p < .01), secondary education (compared to primary education, β = .43, p < .01), never been a smoker (e.g., daily smoker compared to never smokers, β = -.61, p < .001), absence of alcohol consumption (e.g., drinking once a week compared to never drinking, β = -1.09, p < .001), no sports activity (e.g., 2–4 hours per week compared to no sports activity, β = -.60, p < .001), poorer self-rated health (β = .28, p < .001), and more depressive symptoms (β = .05, p < .001). The sensitivity analysis for anxiety symptoms indicated that a preference for solitude was associated with more anxiety symptoms (β = .04, p < .001, not shown in Table 3). The sex-stratified regressions mostly yielded similar results (S1 Appendix).

**Table 3:** Correlates of preference for solitude among the total sample. Findings based on multiple linear regressions.

Independent variables	Preference for solitude–among the total sample
Sex:—Women (Reference category: Men)	0.51***
	(0.09)
• Diverse	-0.52
	(1.32)
Age group: - 30 to 39 years (Reference category: 18 to 29 years)	0.31*
	(0.14)
• 40 to 49 years	0.85***
	(0.14)
• 50 to 59 years	1.12***
	(0.14)
• 60 to 74 years	0.73***
	(0.18)
Marital status:—Divorced (Reference category: Single)	-0.53**
	(0.18)
• Widowed	-0.78**
	(0.25)
• Living together: Married or in partnership	-0.74***
	(0.10)
• Living separately: Married or in a partnership	-0.33+
	(0.20)
Education:—Secondary education (Reference category: Primary education)	0.43**
	(0.14)
• Tertiary education	0.23
	(0.16)
Employment status: Retired (Reference category: Full-time employed)	0.11
	(0.15)
• Other	-0.11
	(0.10)
Region: East Germany (Reference category: West Germany)	-0.20+
	(0.11)
Having a migration background: Yes (Reference category: No)	-0.08
	(0.13)
Smoking:—Yes, daily (never been a smoker)	-0.61***
	(0.11)
• Yes, occasionally	-0.89***
	(0.14)
• No, not anymore	-0.26*
	(0.10)
Alcohol consumption:—Daily (Reference category: Never)	-0.52**
	(0.20)
• Several times a week	-0.97***
	(0.14)
• Once a week	-1.09***
	(0.14)
• One to three times a month	-0.91***
	(0.14)
• Less often	-0.27*
	(0.13)
Frequency of sports activity:—Less than one hour per week (Reference category: No sports activity)	-0.48***
	(0.13)
• 1 to 2 hours per week	-0.44***
	(0.12)
• 2 to 4 hours per week	-0.60***
	(0.13)
• More than 4 hours per week	-0.30*
	(0.14)
Self-rated health (from 1 = very good to 5 = very poor)	0.28***
	(0.06)
Number of chronic conditions (from 0 to 14, higher scores reflect a higher number of chronic conditions)	-0.00
	(0.03)
Depressive symptoms (ranging from 0 to 27, whereby higher scores reflect more depressive symptoms)	0.05***
	(0.01)
Constant	7.32***
	(0.26)
Observations	5,000
R^2^	0.11

Unstandardized beta-coefficients are displayed; robust standard errors in parentheses

*** p<0.001

** p<0.01

* p<0.05

+ p<0.10.

## 4 Discussion

Our study aimed to provide German population norms for the preference for solitude. Furthermore, we explored factors associated with a preference for solitude. This study showed an association between sociodemographic (e.g., sex, age, marital status), lifestyle (e.g., sports activity), as well as health-related factors (e.g., depressive and anxiety symptoms) and the preference for solitude. Sex-stratified regressions produced comparable results. Our study builds on the previous literature that used smaller and selective samples generally focused on adolescents or young adults to provide a better understanding of preference for solitude across adulthood.

Our estimated norms for younger adults are much higher compared to the provided norms by Burger using data from a sample of US college students [1]. However, similar average values were observed in a previous thesis (7.12, SD = 3.16 among emerging adults; 9.02, SD = 2.93 among established adults; 8.72, SD = 3.01 among midlife adults) [19] using data from Amazon MTurk and an undergraduate class (data collection: October to December 2019).

In our study, women had a stronger preference for solitude. In contrast, a former study [20] did not find an association between women and men regarding the preference for solitude among 287 undergraduate students aged 18 to 28 years. This difference might be explained by our focus on general adult population rather than younger adults [20]. The finding is somewhat surprising given that women tend to score higher on prosocial and interpersonal traits [21], and there are no significant sex differences in loneliness among adults [22]. Furthermore, women often have larger social networks than men [23]. However, it may be precisely these larger social networks that can also lead them to prefer opportunities for retreat and be alone. Moreover, women tend to have more family obligations in Germany (e.g., childcare and household work) [24]–factors that may also explain the stronger preference for solitude.

Furthermore, older age groups also had a stronger preference for solitude in our current study. Of interest, this pattern seems opposite to the one found for loneliness, which is lowest in middle-age and higher in the youngest and oldest [25,26]. This may, for example, be an expression of greater family obligations in middle adulthood (e.g., bringing up children, caring for parents) [27]—which at the same time can increase the desire for free time alone. It could also be the desire for autonomous leisure activities after a long working life once in retirement. Similar preferences for solitude depending on age have been found based on 465 young to middle-aged adults using MTurk and a psychology class of undergraduates [14].

Moreover, being single was associated with a stronger preference for solitude (e.g., compared to individuals living together with their spouse or partner). Being single could therefore symbolize the freedom to spend a certain amount of free time alone. This could indicate that being single is an expression of a very free life choice rather than an unsuccessful search for a partner in Germany (see also: [28]). In contrast, individuals who live with their partner or spouse may generally have a greater preference for togetherness (see: [29])—which is ultimately reflected in their living and family situation. In an earlier study, neither marital status nor education was associated with the preference for solitude in different age groups [14]–which may be partly explained by differences in the sample composition. For example, the former study mainly included White/European American adults (mean age of 30.3 years, 61.7% single, 49.7% of the respondents having a college or higher education). Additionally, in this present study, higher education was associated with a stronger preference for solitude, possibly because people with more education place a higher value on self-actualization (e.g., to pursue their own interests) [30]. Such individuals may also find satisfaction more often in activities that they do alone (e.g., reading, creative work) [31].

Not smoking, not drinking alcohol, and not performing sports activities were all associated with a stronger preference for solitude in this present study. It is highly likely that these lifestyle-factors could be perceived as acts of sociability in Germany [32,33]. For example, there are smoking areas in Germany, drinking alcohol is generally socially accepted in Germany (e.g., Oktoberfest), and there are many popular team sports (e.g., soccer, basketball, and handball). Even individual sports often have a social component (e.g., running groups). Dispositional traits are also likely to contribute to these associations: Extroverts, who dislike solitude, are more likely to drink or smoke [34].

Furthermore, adverse health-related factors (i.e., poorer self-rated health and more depressive symptoms) were associated with a stronger preference for solitude. Individuals reporting poor health may suffer pain and discomfort [35], hindering social interactions [36]. Moreover, individuals with perceived poor health may prefer being alone as they may struggle to participate in social activities. They may also favor being alone because they feel like a burden to their friends and relatives [37]. Symptoms of depression include low self-esteem, loss of interest in activities that were previously enjoyable, sleep disturbances leading to fatigue, difficulty concentrating, excessive rumination and negative thoughts, fear of rejection, shame about one’s condition, and a feeling of losing control in social situations. These symptoms may lead individuals with more depressive symptoms to withdraw from social interactions and avoid negative experiences. Ultimately, these factors may lead individuals with more depressive symptoms to have a stronger preference for solitude. Interestingly and of note, sex-stratified regressions showed generally, similar results.

Acknowledgment should be provided for specific strengths and weaknesses of our study. This study represents the first study on German populations for the preference for solitude. Valid tools were used in our study for both the outcome and the independent variables. A very large sample was used, determined by quotas to ensure representation in terms of age, sex, and geographical location. Our research encompassed individuals ranging from 18 to 74 years old. Thus, population norms for individuals ≥ 75 years should be provided in future studies. Moreover, the cross-sectional nature of our data makes it impossible to provide population norms for different periods (e.g., at the peak of the pandemic vs. later stages of the pandemic). Additionally, the possibility of reverse causality cannot be dismissed (e.g., the preference for solitude contributes to changes in lifestyle factors). Furthermore, personality characteristics (e.g., introversion) could not be added to our regression model due to reasons of data availability.

## 5 Conclusion and future research

Norm values provided in this study can be used as a benchmark for comparison with other groups and can guide further research dealing with preferences for solitude. Moreover, knowledge about the correlates of preference for solitude may help to characterize them. This is essential to ensure a good balance between social interaction and being alone.

Norm values in other countries are also highly desirable (e.g., cross-country comparisons). Furthermore, norm values for adolescents may be of interest. Upcoming studies could also explore the moderating and mediating factors. Future research could also explore the consequences of a preference for solitude (e.g., for well-being). To this end, they could explore the enjoyment of solitude and the (perceived) productivity during solitude. Moreover, research could explore the link between the preference for solitude and mental health outcomes in more detail (e.g., when there is an unmet need for the preference for solitude in the job). While we used common analytical approaches in our work, future research could also use more advanced approaches (e.g., machine learning approaches).

## Supporting information

S1 Appendix Correlates of preference for solitude stratified by sex (men; women). Findings based on multiple linear regressions.
(DOCX)
