# Supplementary material for: German population norms of the preference to solitude scale and its correlates
Source: PLoS One. 2024 May 21;19(5):e0303853. doi: 10.1371/journal.pone.0303853 (PMC11108153; doi:10.1371/journal.pone.0303853)
Supplement: S1 Appendix — Findings based on multiple linear regressions. (DOCX) [file pone.0303853.s001.docx]

Appendix 1. Correlates of preference for solitude stratified by sex (men; women). Findings based on multiple linear regressions.

| Independent variables | Preference for solitude – among men | Preference for solitude – among women |
| --- | --- | --- |
|  |  |  |
| Age group: - 30 to 39 years (Reference category: 18 to 29 years) | 0.45* | 0.19 |
|  | (0.21) | (0.18) |
| - 40 to 49 years | 0.88*** | 0.83*** |
|  | (0.22) | (0.19) |
| - 50 to 59 years | 1.14*** | 1.11*** |
|  | (0.22) | (0.19) |
| - 60 to 74 years | 0.82** | 0.66** |
|  | (0.27) | (0.24) |
| Marital status: - Divorced (Reference category: Single) | -0.44 | -0.61* |
|  | (0.28) | (0.25) |
| - Widowed | -0.85+ | -0.75* |
|  | (0.48) | (0.30) |
| - Living together: Married or in partnership | -0.62*** | -0.85*** |
|  | (0.14) | (0.13) |
| - Living separately: Married or in a partnership | -0.55 | -0.24 |
|  | (0.34) | (0.25) |
| Education: - Secondary education (Reference category: Primary education) | 0.26 | 0.62** |
|  | (0.20) | (0.21) |
| - Tertiary education | 0.07 | 0.39+ |
|  | (0.21) | (0.23) |
| Employment status: Retired (Reference category: Full-time employed) | 0.14 | 0.03 |
|  | (0.21) | (0.21) |
| - Other | -0.01 | -0.17 |
|  | (0.17) | (0.13) |
| Region: East Germany (Reference category: West Germany) | -0.16 | -0.26+ |
|  | (0.16) | (0.16) |
| Having a migration background: Yes (Reference category: No) | -0.17 | -0.01 |
|  | (0.19) | (0.17) |
| Smoking: - Yes, daily (never been a smoker) | -0.72*** | -0.50** |
|  | (0.17) | (0.16) |
| - Yes, occasionally | -0.84*** | -0.91*** |
|  | (0.21) | (0.21) |
| - No, not anymore | -0.26+ | -0.28* |
|  | (0.15) | (0.14) |
| Alcohol consumption: - Daily (Reference category: Never) | -0.60* | -0.56+ |
|  | (0.25) | (0.34) |
| - Several times a week | -1.02*** | -1.00*** |
|  | (0.20) | (0.20) |
| - Once a week | -1.27*** | -0.92*** |
|  | (0.21) | (0.19) |
| - One to three times a month | -1.10*** | -0.82*** |
|  | (0.23) | (0.18) |
| - Less often | -0.38+ | -0.18 |
|  | (0.21) | (0.16) |
| Frequency of sports activity: - Less than one hour per week (Reference category: No sports activity) | -0.50** | -0.43** |
|  | (0.19) | (0.17) |
| - 1 to 2 hours per week | -0.58*** | -0.32* |
|  | (0.17) | (0.16) |
| - 2 to 4 hours per week | -0.60** | -0.62** |
|  | (0.19) | (0.19) |
| - More than 4 hours per week | -0.56** | -0.06 |
|  | (0.21) | (0.20) |
| Self-rated health (from 1 = very good to 5 = very poor) | 0.40*** | 0.18* |
|  | (0.09) | (0.09) |
| Number of chronic conditions (from 0 to 14, higher scores reflect a higher number of chronic conditions) | -0.10* | 0.08+ |
|  | (0.05) | (0.04) |
| Depressive symptoms (ranging from 0 to 27, whereby higher scores reflect more depressive symptoms) | 0.05*** | 0.05*** |
|  | (0.01) | (0.01) |
| Constant | 7.40*** | 7.74*** |
|  | (0.37) | (0.36) |
|  |  |  |
| Observations | 2,451 | 2,540 |
| R² | 0.11 | 0.11 |

Unstandardized beta-coefficients are displayed; robust standard errors in parentheses; *** p<0.001, ** p<0.01, * p<0.05, + p<0.10
